# An Opponent Process Cerebellar Asymmetry for Regulating Word Association Priming

**DOI:** 10.1007/s12311-018-0949-y

**Published:** 2018-06-14

**Authors:** Therese M. Gilligan, Robert D. Rafal

**Affiliations:** 1 School of Psychology, Bangor University, Bangor, LL57 2AS UK; 2 Department of Psychological and Brain Sciences, University of Delaware, Newark, DE 19716 USA

**Keywords:** Cerebellum, TMS, Prediction

## Abstract

A consensus has emerged that the cerebellum makes important contributions to a spectrum of linguistic processes, but that the psychobiology of these contributions remains enigmatic (Mariën et al., Cerebellum 13(3):386–410, 2014). One aspect of this enigma arises from the fact that, although the language-dominant left cerebral hemisphere is connected to the right cerebellum, distinctive contributions of the left cerebellar hemisphere have been documented (Murdoch and Whelan, Folia Phoniatr Logop 59:184–9, 2007), but remain poorly understood. Here, we report that neurodisruption of the left and right cerebellar hemispheres have opposite effects on associative word priming in a lexical decision task. Reaction time was measured for decisions on whether a target letter string constituted a word (e.g. bread) or, with equal probability, a pronounceable non-word (e.g. dreab). A prime word was presented for 150 ms before the target and could either, and with equal probability, be related (e.g. BUTTER) or unrelated (TRACTOR). Associative word priming was computed as the reduction in lexical decision RT on trials with related primes. Left cerebellar hemisphere continuous theta-burst transcranial magnetic stimulation (TMS) decreased, and right hemisphere stimulation increased, priming. The results suggest that the cerebellum contributes to predictive sequential processing, in this case language, through an opponent process mechanism coordinated by both cerebellar hemispheres.

## Introduction

Leiner et al. [1] were early advocates of the likely contributions of the cerebellum to language. Subsequent anatomical and clinical observations have provided strong support for a role of the cerebellum in both cognitive and affective domains [2] with the heuristic hypothesis that cerebellar damage in humans results in a ‘dysmetria of thought’ [3]—the idea that there is a disturbance in the coordination of thought analogous to that seen with movements.

Indeed, the wide range of mild linguistic deficits documented following cerebellar damage (e.g. impairments in lexical access, phonological and semantic verbal fluency, syntax processing, reading, writing and speech) has led to the idea of cerebellar aphasia gaining ground and an agreement that the problems relate to control of language processes rather than an impairment in language components [4–6]. Nonetheless, the psychobiology of cerebellar contributions to language remains enigmatic [7].

Recent interest has focused on the potential role of the cerebellum in providing a prediction mechanism that facilitates not only language production but also language comprehension (for reviews, see [8–10]). Here, we focus on two studies of particular interest. Lesage et al. [11] employed a paradigm, wherein they recorded eye movements of people listening to sentences, while viewing four pictures, one target and three distracters, at the corners of an imaginary square. The target was a picture of an object named at the end of the sentence. The sentences were either predictive or non-predictive. For example, in a predictive sentence, ‘the man will sail the boat’, the pictures could be a boat/bird/car/house, and a non-predictive sentence might be ‘the man will watch the boat’, with the same set of pictures. When a sentence was predictive, participants made anticipatory eye movements towards the target (boat). The reduced latency of the first saccade towards the target picture when sentences are predictive vs. non-predictive provides a measure of predictive language priming. Participants were tested before and after offline 1 Hz repetitive transcranial magnetic stimulation (TMS) of the right cerebellar hemisphere (1 cm below, 3 cm to the right of inion) for 10 min. TMS reduced the effect of prediction on saccade latencies to target pictures.

Using a different offline repetitive TMS procedure (continuous theta-burst (cTBS)), Argyropoulos [12] examined the effects of right medial (1 cm below, 1 cm to the right of inion) cerebellar hemisphere disruption in a word association priming paradigm in which prime words were phrasally/temporally (e.g. pigeon-HOLE) or categorically (e.g. penny-COIN) related to the target. Disruption of the right cerebellum resulted in an increase in phrasal associative word priming but had no effect on categorical priming. That is, the observed change was in the condition in which there was a temporal relationship between the words. In an extension of that study, disruption of the lateral right (approx. 10 cm right of inion) cerebellar hemisphere resulted in enhancement of semantic noun-to-verb priming based on association (e.g. ‘soap-cleaning’), but had no effect of priming based on categorical similarity (e.g. ‘robbery-stealing’) [13].

Reflecting those findings, some authors have called upon the work of Ivry and Richardson [14] to propose that cerebellar-induced linguistic deficits are due to a timing disorder (e.g. [14]). Building on studies of ataxic dysarthria (a disruption of speech articulation and prosody), Ackermann [15] has specified a role for the cerebellum in the ‘temporal organisation’ of speech, an argument that has been expanded upon by Kotz and Schwartze [16]. While some authors do support a role for the cerebellum in temporal and spatial sequencing of activities [17], others give more weight to its function as a comparator of temporally accurate predictions or internal models [18] with the actual sensory feedback of action (reafference) [19]. It has, however, been pointed out that all predictions contain a ‘what’ and a ‘when’ element (to some degree). Moberget and Ivry [9] argue that for now, the evidence for a predictive role of the cerebellum in language is predominated by the when element. Whereas, Argyropoulos [8] argues that in fact the evidence for a predictive role of the cerebellum in the non-motor aspects of language processing to date remains inconclusive.

Both the Lesage et al. [11] and the Argyropoulos [12] experiments mentioned above employed TMS disruption of the right cerebellum. The former resulted in a reduced benefit of prediction in the visual world experiment, whereas, paradoxically, in the latter, the priming effects of prediction were increased. While there were several differences between these two studies, one salient difference was the interval between the processing of priming and target words. Given the posited role of the cerebellum in timing, particularly in the millisecond range, we wondered whether one of the reasons behind the apparently conflicting results might be related to the fact that both TMS studies used different intervals between the presentation of the prime and target words (longer and shorter, respectively). In the current experiment, we thus focused on short intervals (when we might assume processing of the prime word was still ongoing [20]) but used the stimulation coordinates of the Lesage et al. [11] study with the expectation of increased predictive priming following neurodisruption of the right cerebellum.

Both TMS studies [11, 12] stimulated the right cerebellar hemisphere only. However, a meta-analysis of neuroimaging studies has implicated both cerebellar hemispheres in language [21], and both left and right cerebellar damage have been implicated in language deficits (for reviews, see [21, 22]). We could identify only two TMS language studies that have included left cerebellar stimulation. Allen-Walker and coworkers [19] found evidence of left cerebellar involvement in backward priming at short intervals, whereas Runnqvist and colleagues [23] found no evidence for left cerebellar involvement in a speech production task. Elsewhere, a study that imaged metabolic changes before and after TMS of the left cerebellum (1 cm below inion and 3 cm lateral) noted changes bilaterally in the superior and middle temporal gyri [24]. Taken together with the increasing evidence and acceptance for bi-lateral cerebro-cortical involvement in language (e.g. [25, 26]), we expected an effect of left cerebellar TMS on predictive priming.

Here, we report that TMS disruption of the right and left cerebellar hemispheres with sub-threshold cTBS has opposite effects on associative word priming in which there is a forward (i.e. predictive) relationship between prime and target words in a lexical decision task. We relate our findings to evidence from neuropsychology of aphasia and hypotheses of cerebellar function and suggest that any cerebellar role in predictive sequential processing is mediated by a coordinated opponent process mechanism involving both hemispheres.

## Methods

In a mixed group design, automatic word association priming effects were measured in a lexical decision task before and after 40 s of cTBS. One group of participants was stimulated over the left cerebellum and another group over the right cerebellum. Participants were additionally stimulated at a vertex control site, with half stimulated first over the cerebellum or vertex in sessions 1 week apart.

### Participants

Forty-one self-reported neurologically healthy participants, 21 women (mean age 23.4 years, SD = 5.5) were recruited from the university community. All were right-handed [27] with normal or corrected-to-normal vision, non-dyslexic and mono-lingual English speaking. Bangor University Ethics Committee approved the research, which was conducted in concordance with the Declaration of Helsinki, and a health screen/medical history questionnaire was employed to ensure that individuals with a medical history (of epilepsy, brain disease, migraine, or use of psychotropic medication) that would contraindicate brain stimulation were excluded. One group of participants received right (*n* = 21) and the other left (*n* = 20) cerebellar stimulation; participants were randomly assigned to a group. Independent *t* tests revealed no significant differences between the groups for age, *p* = .119 (non-parametric, Mann-Whitney *U* test) or handedness, *p* = .855 (non-parametric, Mann-Whitney *U* test). The gender balance was 8/13 m/f and 12/8 m/f in the right and left group, respectively. Each group was stimulated over a vertex control site in a separate session. Site order (cerebellum/vertex) was counterbalanced with sessions 1 week apart. Decision on sample size was based on the Lesage et al. [11] study.

### Brain Stimulation

cTBS [28] was administered using a Magstim Super-Rapid stimulator with a 70-mm figure-of-eight coil. Stimulator output was individually set to 80% of each individual’s resting motor cortex threshold (mean 59.2% of maximum stimulator output (SD = 9.8)); stimulation intensity was chosen based on a study that showed disruption of classic eye blink conditioning using these stimulation parameters [29]. The coil paddle pointed posteriorly for vertex and superiorly for cerebellar stimulation. The location of cerebellar stimulation (1 cm below and 3 cm lateral to the inion) corresponded to that used in the Lesage et al. [11] study. The use of generic scalp-based landmarks, as opposed to the use of neuronavigational apparatus, will have introduced some variability as well as an inability to specify the precise stimulation site in each individual. However, the most likely areas to have been stimulated using these scalp landmarks include portions of HVI, HVIIa Crus I and HVIIa Crus II [10, 11, 30].

Continuous TBS induces inhibitory effects; it has been shown to reduce cerebellar excitability and modulate its output to contralateral interconnected cortical areas [31]. Cerebellar cTBS is thought to interfere with cerebellar function by inducing a long-term depression-like effect. It is likely that only the cerebellar cortex was directly stimulated by the cTBS but it cannot be ruled out that deep cerebellar nuclei or extracerebellar structures (e.g. olivary nuclei) were subject to secondary effects [29].

### Lexical Decision Task

A stimulus that activates the meaning of a word (e.g. salt) facilitates subsequent processing of other words with which it is often associated (e.g. pepper) [32]. This word association priming effect can be measured experimentally as a reduction in latency to recognise a target letter string as a word in a lexical decision task**.** Word association priming is contingent on how likely one word will bring another to mind based, for example (and as designed in this study), on the likelihood that the two words will occur in temporal contiguity. In a typical lexical decision task, the dependent variable is the time to make a decision whether a target letter string is a word (e.g. bread) or, with equal probability, a pronounceable non-word (e.g. dreab). The target string is preceded by a prime word that is either associated with the target word (e.g. butter) or, with equal probability, not associated (e.g. tractor). The associated prime condition results in shorter response times (RT) to make the lexical decision.

### Procedure

Experimental stimuli were pairs of letter strings sequentially presented at the centre of a CRT monitor at eye level 57 cm in front of the participant. Presentation of stimuli and recording of responses was controlled using E-Prime software on a Windows-based personal computer. Participants were asked to read on screen a series of pairs of letter strings and to respond only to the second letter string by indicating, as quickly and as accurately as possible, whether or not it was an English word.

Participants started with a long practice block of 64 trials to check understanding and to minimise practice effects across experimental sessions. Participants recorded their answers by pressing one of two keypad buttons using the middle and index fingers of their left/right hand; button allocation was counter-balanced across participants.

Each experimental block consisted of two lists of 80 trials each. The presence of two lists allowed for a short break (c30 s) during the block. Each trial began with the presentation of a fixation + for 500 ms followed by the prime word in uppercase for 150 ms, followed by a blank screen lasting 100 ms (± 50 ms) and then the target letter string in lowercase. Targets remained on screen until the participant responded. The inter-trial interval was 1500 ms, and the order of stimuli presentation was pseudo-randomised. Use of the stimuli blocks before or after stimulation was counter-balanced across participants. This controlled for any differences in the stimuli beyond forward association strength (see “Stimuli” section). In total, each participant was presented 320 word pairs over two blocks (pre and post TMS) during an experimental session.

Participants completed the practice block, two pre-stimulation blocks, received rTMS, waited 5–6 min, and then completed two post-stimulation blocks. Participants receiving left cerebellar stimulation were instructed to use their right hand for the task across both sessions (vertex and cerebellar stimulation), and vice versa.

### Stimuli

Stimuli consisted of four lists, two for pre-TMS use and two for post-TMS use, each containing 20 related and 20 unrelated prime-target word pairs, and 40 prime-non-word pairs; this resulted in an equal probability mix ratio. While a 25% probability of a related pair is relatively high and may introduce a conscious strategic element to the lexical decision [33], proportionality effects are understood to have a diminished role at short stimulus onset asynchrony (< 400 ms) as is used here [34]. Stimuli examples are *SALT-pepper* (related), *GIRL-stamp* (unrelated) and *NIGHT-henost* (non-word). A practice stimuli set consisted of 16/16/32 pairs, respectively.

The Semantic Priming Project database [35] was mined for pairs based on the following prime characteristics: forward associative strength 0.4 and above, word length 3–10, RT, and automatic priming effect size. The related pairs in this database were formed from on the basis of ‘the first thing that comes to mind when you see word “xxx”’. Thus, the forward associative strength criterion was key; the aim for the associated pairs was to produce pairs that were related over time, i.e. temporally/sequentially predictive. This search resulted in 395 prime-target pairs. The pairs were searched for any repetitions across prime or target; the pair containing the repetition with the lowest forward associative strength and/or priming effect at short SOA was eliminated, resulting in 349 pairs. A further 20 pairs were removed due to potential cultural differences between US and UK English. The remaining pairs from this master list were ordered based on size of priming effect, and the list splits in two: one with positive priming (232 pairs) and one with negative priming (97 pairs).

The order of the positive priming master list was randomised, and four groups of 20 pairs were sequentially chosen and assigned to the different stimuli lists as ‘related pairs’. Then, another four groups of 20 pairs were sequentially chosen from the remainder of the positive priming master list and assigned to the stimuli lists as ‘unrelated pairs’. All these eight lists were then compared and adjusted, through swapping pairs, so that on visual inspection, they were comparable across forward associative strength, word length, word frequency, lexical decision task RT, automatic priming effect size, and mix of relationship types. The pairs assigned as ‘unrelated’ were rotated to make up unrelated pairs, checked for unexpected forward or backward priming, and adjusted accordingly.

The remaining pairs from the original master list (all having a positive forward associative strength but not all achieving a positive priming effect) were randomised in order. Sequential groups of 40 pairs were allocated to each of the four stimuli lists as ‘non-word pairs’. These sub-lists were then visually compared and adjusted as before. Finally, the non-words were created by changing the position of one or two letters in the target to create a pronounceable non-word (avoiding the creation of a pseudo-homophone) that was also orthographically and phonologically plausible; these non-words were then rotated for pairing with a new prime [36].

While the origin of the database was based on free associations (i.e. what comes to mind first when presented with word ‘xxx’), as its name suggests, the Semantic Priming Project database [35] includes semantic relationships. In the stimuli set used here, a significantly larger semantic/categorical similarity was present in the related (*M* = 0.360) compared to the unrelated pairs (*M* = 0.117) as measured by the WordNet::Similarity tool (*t*(89.9) = 6.42, *p* < .001) [37]. However, as described in the next sections, the results are consistent with the findings of Argyropoulos [12] for phrasally associated word pairs and of Argyropoulos [13] for categorical associations. Thus, we assume our related pairs to have a sufficient forward association relation to facilitate temporal/sequential predictions.

## Results

After excluding errors (< 0.04%) and trials with RTs < 200 ms or > 1500 ms RT (< 0.001%), data were submitted to a mixed repeated measure ANOVA with group (left or right cerebellar stimulation) as a between subject factor and within subject factors of prime relatedness (associated or unrelated) × time (pre, post cTBS) × site (cerebellar or vertex). RT was the dependent variable; changes in it passed tests of normality (*D*), skew and kurtosis, allowing parametric analysis. Follow-up analyses were conducted with Duncan’s test.

Table 1 reports mean RTs in each condition. RT was shorter when primes were associated than when primes were unrelated (*F*[1, 39] = 229.2, *p* < .001).

**Table 1:** Mean RT (ms with SE in parentheses) in each condition for the group that received right cerebellar stimulation (top) and left cerebellar stimulation (bottom)

Site	Before cTBS		After cTBS	
	Associated prime	Unrelated prime	Associated prime	Unrelated prime
Vertex	504 (12)	549 (13)	500 (13)	537 (14)
Right	506 (13)	542 (14)	508 (16)	556 (15)
Vertex	515 (17)	542 (19)	516 (19)	549 (21)
Left	509 (19)	547 (19)	513 (20)	538 (20)

**Fig. 1:**
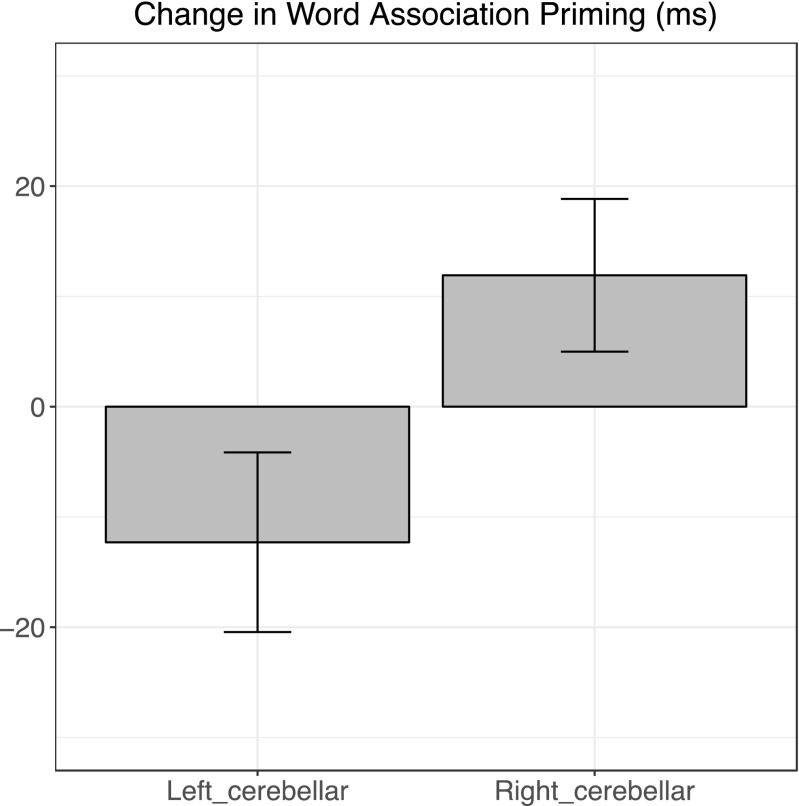
Change in mean word association priming effect in ms (RT for unrelated minus related prime conditions) following 40 s of sub-threshold continuous theta-burst rTMS to either right or left cerebellum. Error bars are SE

Cerebellar stimulation caused a change in word association priming compared to vertex stimulation, and this change differed depending on the cerebellar hemisphere stimulated as revealed by a significant four-way interaction of group (right cerebellar vs. left cerebellar) × site (cerebellar vs. vertex) × time (pre vs. post cTBS) × prime relatedness (associated vs. unrelated) (*F*[1, 39] = 12.7, *p* < .001). The site (cerebellum vs. vertex) × time (pre vs. post cTBS) × prime relatedness (associated vs. unrelated) interaction was reliable in both right (*F*I[1, 20] = 6.2 *p =* .022) and left (*F*I[1, 19] = 6.7, *p =* .018) cerebellar stimulation groups**.** Word association priming increased after right (*p* = .049) and decreased after left (*p* = .046) cerebellar stimulation (Fig. 1)*.* There was no effect of stimulation of the vertex control site in either group.

The near absence of inaccurate answers precluded analysis for effects of cTBS on accuracy or speed/accuracy trade-offs. There was a trend towards a significant effect of time (pre, post) on the RT for non-words (*F*[1, 39] = 3.80, *p* = .058), with non-words responded to faster following cTBS (*M* = 540) compared to before it (*M* = 546). There was no significant interaction of time × site (*F*[1, 39] = 0.73, *p* = .399) or of time × site × group (*F*[1, 39] = 2.29, *p* = .138). This indicates that cerebellar cTBS did not affect any reading-related processes or motor processes for the expression of lexical decisions.

## Discussion

We found that word association priming increased following right cerebellar stimulation and decreased following left cerebellar stimulation. Specifically, disruption of the right cerebellum with cTBS resulted in an increase in magnitude of word association priming when there was a short interval between presentation of prime and target words. The novel result of this experiment is that left cerebellar hemisphere disruption led to a decrease in word association priming. To our knowledge, this is the first demonstration of such an opponent-process cerebellar asymmetry in any domain.

Building on a postulated role of the cerebellum in neural prediction (as set out in the introduction), the findings offer preliminary evidence for a hypothesis specifying the contribution of the cerebellum to dynamic sequential predictions. The current study examined the role of the cerebellum in language, a cognitive process in which dynamic sequential predictions are critical. We will first present evidence from the literature that word association priming is normally subject to early inhibition such that the meaning of potentially upcoming related words is not made available to cortical networks engaged in language processing until extraction of the meaning of the priming word is completed. We hypothesise that together, the cerebellar hemispheres trigger the availability of these predictions, with each cerebellar hemisphere differentially mediating the early inhibition of words temporally/sequentially associated to the prime.

### Contrast with the Effects of Cerebral Hemisphere Lesions on Word Priming

As a starting point for consideration of this hypothesis, let us contrast the current findings with the effects of left and right cerebral hemisphere lesions on word priming. Henik and colleagues [38] reported that left (anterior and posterior) hemispheric lesions reduced priming effects in a lexical decision task (mean − 3 ms) compared to healthy controls (mean 20 ms) whereas right cerebral hemisphere lesions increased priming effects (mean 64 ms). The reduced priming in patients with left hemisphere lesions could not be attributed to a failure of lexical processing since identity priming (e.g. bread-BREAD) was preserved and comparable to controls.

One account of these results is that left hemisphere lesions reduce the spreading activation of words related to the priming word. However, many left hemisphere-lesioned patients with word finding difficulties make naming errors that are associatively related to the word they are trying to retrieve (semantic paraphasia; e.g. substituting fork for knife). Thus, there is a paradox. The phenomenon of semantic paraphasia implies that patients with word finding difficulties do activate associated words and that they compete with the word they are attempting to retrieve. Yet left hemisphere lesions tend to reduce word association priming in a lexical decision task. One possibility is that this priming is not abolished by left hemisphere lesions, but rather that it is *dysregulated*; that is, there is insufficient inhibition of related words before complete retrieval of the word currently being processed.

Consistent with this explanation, Bushell [39] demonstrated negative word priming in patients with left hemisphere lesions (selected for having Broca’s aphasia). One consistent finding in word priming research is that the size of the priming effect increases as the proportion of related (vs. unrelated) primes increases [40, 41]. Bushell replicated this effect in control participants; however, strikingly in patients, word priming *decreased* as the proportion of prime-target relatedness increased, and in blocks where there was a high probability that the target word would be related to the prime, patients demonstrated *negative* priming. By contrast, identity priming in patients increased as a proportion of identical prime-target trials increased, just like controls.

Bushell [39] interpreted her findings in aphasic patients as being consistent with the centre-surround account of Carr and Dagenbach [42]. They had shown that when participants were required to deeply process a prime word, the perception of which was rendered difficult by masking, negative semantic priming was observed; i.e. RT was longer on trials where the prime and target words were related. They inferred that word retrieval (at least under circumstances where semantic codes are weakly activated) involves a neural mechanism to ‘enhance activation of sought for codes and to inhibit nearby codes stored in a semantic network’. More recent research has validated their supposition that negative priming results from a centre-surround mechanism [43, 44].

Bushell [39] argued that because aphasic patients have difficulty processing words, they must inhibit activation of related words until processing of the prime word has been completed. When the expectation that the target word will be related to the prime is higher (as is the case when the relatedness proportion increases), related words are more strongly activated—and thus require more inhibition, resulting in negative priming. Thus viewed, decreased priming in aphasia may be construed as compensatory mechanism that suppresses paraphasic errors.

### Cerebellar Contributions to Predictive Word Priming

In contrast with the effects of cerebral hemisphere lesions, cerebellar neurodisruption (we assume cTBS to have disruptive inhibitory effects, see “Methods” section) has the opposite effect on word association priming: neurodisruption of the right cerebellum (which is connected to the left cerebral hemisphere) increases associative word priming ([12] and the current experiment), whereas neurodisruption of the left cerebellum (which is connected to the right cerebral hemisphere) decreases associative word priming. One interpretation of the opposite effects of cerebral and cerebellar hemisphere disruption is that the right cerebellar hemisphere is involved in triggering the release of primed words from early inhibitory effects (which Henik et al. [38] showed to be enhanced in patients with left hemisphere lesions who, therefore, demonstrate decreased associative priming), and the left cerebellum is involved in triggering the facilitation of early inhibition of priming (which Henik et al. [38] showed to be absent in patients with right cerebral hemisphere lesions relative to controls, and who therefore show increased associative priming.)

We acknowledge this to be a new interpretation of the role of the cerebellum in sequential processing. Thus, we next relate it to findings on cerebellar involvement in scheduling activities and expand on the need for precisely timed on-off scheduling in language. We finish by outlining future research needed to marshal support for the interpretation.

### Cerebellum: Timing and Prediction

Keele [45] introduced the concept of the ‘motor program’ as an abstract representation of an intended movement, containing not only the goal of the action but also the possible processes necessary to implement it. The concept implies the program of a motor sequence prepares not only the order of the sequence but also the timing such that while one element of the sequence is being activated, the next is inhibited until its predecessor is completed. The sequence length effect reflects real-time demands for the setup of timed response schedules for individual elements of a motor program prior to its execution [46]; the longer the sequence, the longer it takes to set it up [47]. It has been shown that the cerebellum plays a role in the *scheduling* of pre-programmed, fluent motor sequences [48]. Cerebellar patients and healthy controls were tested using sequences of finger movements of varying lengths. The sequence length effect was reduced in patients with moderate, and was absent in patients with severe, cerebellar-induced motor disability—thus demonstrating an impaired ability to schedule a sequence of successive motor events before movement onset.

The involvement of the cerebellum in time sensitive activities has been well described: from Braitenberg’s [49] suggestion that it functions as a biological clock in the millisecond range, to imaging studies supporting that notion in healthy participants [50, 51], and to studies showing impaired timing in motor and perceptual tasks in the presence of cerebellar lesions, both real [52–55] and virtual [56]. This cerebellar function(s) of scheduling and timing can also account for findings in the word priming literature.

Lexical access in speech production proceeds at a rate of about two to three words per second and is encoded phonologically at a rate of about 15 speech sounds per second [57]. Necessarily, then, language requires dynamic predictive processes to meet these challenges. Word priming affords one potential mechanism to meet these challenges. However, while the priming of related words has the potential to facilitate the dynamic efficiency that permits fluent production and perception, effective priming must be precisely timed. While each word is being processed, related words begin to be activated, but their availability to cortical networks engaged in production and comprehension must be inhibited until the appropriate ‘clock pulse’. If a primed word is activated too soon, it can compete with its prime, delaying access to the prime or causing naming errors. Thus, the availability of primed words for sequential language processes must be modulated by brain mechanisms that facilitate *and inhibit* it with a temporal precision needed for both accuracy and fluency [16].

We therefore interpret the increase in word association priming after right cerebellar neurodisruption, not as an improvement, but as a *dysregulation* of timing resulting in premature release from inhibition of related word meanings. We propose that the cerebellum is critical in timing the availability of predictions, with the right cerebellum triggering their release from inhibition after the processing of the prime word (or the current event in any sequence) is complete, whereas the left cerebellum triggers the early inhibition of predictions while the prime (current event) is being processed.

This interpretation suggests that the cerebellum has a supportive role within the wider linguistic network that includes the prefrontal and temporal cortices to which it is connected [58, 59]. It is generally assumed in research in which brain stimulation is presumed to induce a ‘virtual lesion’, that any behavioural impairment implicates the stimulated region as contributing some necessary computation supporting that behaviour. An alternative account is that the disrupted region does not necessarily contribute to such functions *sui generis*, but rather that stimulation has a remote effect on some other functionally critical region with which it is connected. On this account, the cerebellum could be part of a neural circuit, including prefrontal cortex that is responsible for word priming effects. We are proposing that the cerebellum does make a specific computational contribution to priming by providing timing signals necessary to optimise the benefits of predictive signals.

In summary, we hypothesise that both facilitation and inhibition of sequential priming are regulated by the cerebellum and that the right and left cerebellar hemispheres function as an opponent process in which the left cerebellum inhibits and the right facilitates sequential priming*.*

This hypothesis makes specific predictions to be tested in future research:

1. That prime words are less efficiently processed when the right cerebellar hemisphere is stimulated. The current study did not incorporate any measures of how efficiently the priming words had been processed. Future research could include testing of memory for prime words after completion of the post-stimulation lexical decision task to test the prediction that recall of prime words will be better after left cerebellar stimulation than right cerebellar stimulation.
2. The pattern observed in the current study, of increased word association priming with right cerebellar disruption (and decreased word association priming with left cerebellar stimulation), will occur only when there is a brief delay between prime and target (while the prime is still being processed). For longer delays (e.g. stimulus onset asynchronies (SOAs) between prime and target words > 600 ms), this pattern will not be present and, indeed, based on the effects of right cerebellar TMS in the Visual World Paradigm, would actually be expected to be the reverse: a decrease in word association priming after left and an increase word association priming after right cerebellar theta-burst stimulation.
  While this specific prediction has not yet been tested explicitly, it is interesting to note that the only other study of word association priming in which the left cerebral hemisphere was stimulated reported, in contrast with our findings, an increase in priming [19]. That experiment, like ours, employed a short interval (250 ms) between prime and target word. However, Allen-Walker et al. [19] examined backward priming in which the prime word was associated with the target word, but did not predict it. For example, in the pair dog-BONE, dog is predictive of bone; whereas the two words are much less likely to appear in the reverse order bone followed by dog. In this latter case of backward priming, the lexical decision on the target word can be aided by retrieving the prime word from episodic memory or as Allen-Walker et al. [19] suggested by a feedback loop. Such post lexical processing can begin only after the meanings of both prime and target words have been successfully extracted. Thus, although the experiment employed a short prime-target interval, the process affected by cTBS was one that operates quite late, well after there is any need to protect the prime word from inhibition by related words. Consistent with our prediction, stimulation of the left cerebellar hemisphere in this backward priming experiment resulted in an increase in priming.
3. If the hypothesis is correct (that a reduction of early inhibition of predictions results from disruption of the right cerebellum and thus interferes with accessing the prime word), this could be tested by measuring the effects of cerebellar cTBS on identity priming. If right cerebellar disruption disinhibits activation of related words before the prime word is fully processed, we would expect that right cerebellar cTBS will reduce the RT benefits of identity priming (again compared to an unrelated prime condition), whereas left cerebellar disruption is predicted to increase identity priming.
4. Note, crucially, that the hypothesis outlined here does not invoke a role of the cerebellum in word processing per se, but rather a more general control function that regulates timing the release of predictions in sequential behaviour. Thus, we would expect similar effects of cerebellar stimulation on sequential predictions that do not involve words such as picture priming [60], and predictive sequencing of simple tones [61].
5. Finally, the current paradigm could be re-run with some improvements: to address concerns regarding the ‘purity’ of the current stimuli a set of forward-phrasal related pairs that exclude categorical/semantic relations and with a probability ratio < 20% could be tested. Additionally, the use of neuronavigation tools would improve the ability to target the posterolateral cerebellum with cTBS. We would expect the current results to be replicated and produce opposite effects for each cerebellar hemisphere indicative of an opponent process mechanism underlying predictive sequential processing.
